# Association between hospital accreditation and healthcare providers’ perceptions of patient safety culture: a longitudinal study in a healthcare network in Brazil

**DOI:** 10.1186/s13584-025-00690-8

**Published:** 2025-06-04

**Authors:** Helidea de Oliveira Lima, Leopoldo Muniz da Silva, Leandro Reis Tavares, Ana Claudia Lopes Fernandes de Araújo, Laise Pereira Moreira, Vanessa de Melo Silva Torres, Fabiana Nogueira de Oliveira, Anthony M.-H. Ho, Deborah Simões, Glenio B. Mizubuti, Joaquim Edson Vieira

**Affiliations:** 1 IDOR– D’Or Institute for Research and Education - Rede D’Or, São Paulo, Brazil; 2 Department of Patient Safety and Quality Management, São Luiz Hospital (Itaim), Rede D´Or, Rua Alceu de Campos Rodrigues, 229, São Paulo, SP Brazil; 3 Department of Patient Safety and Quality Management, Vila Nova Star, Rede D´Or, São Paulo, SP Brazil; 4 Department of Anesthesiology and Perioperative Medicine, Queen’s University, Kingston, ON Canada; 5 Postgraduate Program in Anesthesiology, Surgical Sciences, and Perioperative Medicine, Faculdade de Medicina FMUSP, Universidade de São Paulo, São Paulo, SP Brazil

**Keywords:** Accreditation, Crew resource management, Healthcare, Organizational culture, Patient safety, Surveys and questionnaires

## Abstract

**Background:**

Enhancing security and dependability of health systems necessitates resource allocation, a well-defined infrastructure, and a steadfast commitment to ensuring its safety and stability over time. This study aimed to assess changes in patient safety culture over time (2014–2022) within a network of private hospitals in Brazil and to examine its association with the hospital accreditation process. The study utilized the Hospital Survey on Patient Safety Culture (HSOPSC) to measure healthcare professionals’ perceptions of patient safety culture.

**Methods:**

The HSOPSC questionnaire was distributed to 71 hospitals between 2014 and 2022 with 259,268 responders. Hospitals were classified as accredited (AH) or non-accredited (NAH). A linear mixed-effects regression model was used to analyze the trend of dimension scores over time, accounting for both fixed and random effects to accommodate within-hospital correlations and variations across time points.

**Results:**

Out of 12 dimensions analysed, 11 significantly improved, and one (“frequency of reported events”) remained unchanged over time (*p* = 0.84). Two dimensions had < 50% positive responses: “communication openness” (47.13% [38.19–58.73]) and “nonpunitive response to errors” (41.24% [34.13–51.98]). Safety culture improved among AH across all, but “frequency of reported events” (*p* = 0.12), dimensions. Among NAH, “frequency of reported events” decreased over time (*p* = 0.008) while other dimensions remained unchanged.

**Conclusion:**

Our results suggest an improvement in patient safety culture within this network of private hospitals in Brazil from 2014 to 2022. While accreditation appears to be associated with fostering a culture of safety over time, our study does not establish a causal relationship. Additionally, non-accredited hospitals tended to report fewer adverse events, which may indicate underreporting and missed opportunities for healthcare system improvement through adverse event analysis.

**Supplementary Information:**

The online version contains supplementary material available at 10.1186/s13584-025-00690-8.

## Introduction

Patient safety is a fundamental pillar of healthcare quality and a prerequisite for delivering effective and reliable care. Achieving a high level of patient safety requires a strong institutional culture in which all healthcare professionals are committed to promoting excellence. In recognition of this need, the Brazilian Ministry of Health launched the National Patient Safety Program (NPSP) in 2013, aiming to improve healthcare quality across the country’s two-tier healthcare system [1] This system consists of the publicly funded Unified Health System and the privately funded Private Health sector, which serves approximately 24.5% of the population of over 211 million people [2, 3]. In response to this national initiative, hospitals in both sectors established Patient Safety Centers to strengthen safety culture and implement quality improvement strategies. While studies have explored the evolution of patient safety culture in public Brazilian hospitals following the implementation of the NPSP, its impact within private hospitals remains underexplored [4, 5].

A key component of the NPSP is its emphasis on hospital accreditation, which has long been considered a strategy for enhancing healthcare quality and patient safety. However, the actual influence of accreditation on hospital performance and patient outcomes remains uncertain [6–8].

Although accreditation is associated with increased compliance with safety protocols and the implementation of best practices, its direct effect on reducing patient morbidity and mortality is still debated [9, 10]. Some studies suggest that accreditation fosters a culture of continuous quality improvement, yet there is no definitive evidence linking accreditation to sustained behavioral changes among healthcare professionals or to measurable improvements in patient outcomes [8, 11, 12]. Achieving a true transformation in safety culture requires more than compliance with external standards; it demands a fundamental shift in attitudes, behaviors, and organizational commitment to safety [6, 13]. Despite the lack of conclusive causal evidence, hospital accreditation is often seen as a catalyst for performance improvement and patient safety initiatives [6, 14].

Given the widespread implementation of accreditation in Brazil’s private hospital sector, we hypothesized that accredited hospitals would demonstrate greater improvements in patient safety culture over time compared to non-accredited hospitals. Specifically, we expected accreditation to be associated with higher adherence to safety practices and improved staff perceptions of safety culture. This study aimed to assess changes in patient safety culture over time (2014–2022) within a network of private hospitals in Brazil and to examine its association with the hospital accreditation process. The study utilized the Hospital Survey on Patient Safety Culture (HSOPSC) Version 1.0 [15, 16] to measure healthcare professionals’ perceptions of patient safety culture.

## Methods

The Hospital Survey on Patient Safety Culture (HSOPSC) Version 1.0 [15, 16] is a validated instrument widely used to evaluate perceptions of safety culture among healthcare professionals. This survey measures multiple dimensions of patient safety culture, including leadership commitment to safety, teamwork, communication, incident reporting, and overall safety perceptions. Examples of questionnaire items include: *‘Hospital management provides a work climate that promotes patient safety’* (leadership commitment), *‘Patient safety is never sacrificed to get more work done’* (staff perception of safety culture), and *‘Staff feel free to openly discuss errors and safety concerns’* (willingness to report safety issues). This retrospective cohort study was conducted using the HSOPSC adapted to Portuguese and for use in the Brazilian context [16] from September 2014 (the year following NPSP implementation) to September 2022. The study population consisted of professionals working in 71 private hospitals in Brazil, distributed across five macro geographic regions, i.e., North, Northeast, Central-West, Southeast, and South. All hospitals in this study were general hospitals with intensive care unit (ICU) beds, surgical centres, and emergency departments.

The HSOPSC aims to capture the perception of culture through 12 dimensions: (1) communication openness; (2) feedback and communication about errors; (3) handoffs and transitions; (4) management support for patient safety; (5) nonpunitive response to errors; (6) organizational learning; (7) overall perception of patient safety; (8) staffing; (9) supervisor/manager expectations and actions promoting safety; (10) teamwork across units; (11) teamwork within units; and (12) frequency of events reported. These dimensions address critical aspects related to the organization’s beliefs and norms, values, communication, leadership, management, and reporting of adverse events (10). The combined dimensions amount to a total of 42 items out of which 33 were answered using a five-point Likert scale (indicating the degree of agreement from “totally disagree” to “totally agree”), and the remaining 9 items used a 5-point frequency scale (ranging from “never” to “always”). The patient safety rating was categorised as excellent, very good, good, fair, or poor. The culture of safety is assessed from the staff’s perspective.

A web-based platform was developed to administer the HSOPSC survey. Participants completed the questionnaire online at their preferred location and time. Surveys were conducted annually. Hospital participation was voluntary. The survey was open to physicians, nurses, medical technicians, and pharmaceutical, administrative, and rehabilitation staff. Apart from hospital’s name, no identifying information that would reveal the survey respondents was collected.

The assessment of patient safety culture using the HSOPSC involved calculating the percentage of positive responses for each positively written dimension, as per the following formula: [number of positive responses to the items within the assessed dimension ÷ total number of valid responses to the items within the assessed dimension (positive, neutral and negative data)] × 100%. For example, a positively worded item such as ‘Hospital management provides a work climate that promotes patient safety’ was considered positive if respondents answered ‘Strongly Agree’ or ‘Agree’. In contrast, a negatively worded item like ‘Staff are afraid to report patient safety issues’ was reverse-scored, meaning disagreement (‘Strongly Disagree’ or ‘Disagree’) was considered a positive response. The percentage of negative responses is similarly calculated.

The positive/negative response rates reflect attitude toward patient safety culture and aids in identifying strong/weak areas in patient safety. “Strong areas of patient safety” include items with ≥ 75% positive responses for items written positively or ≥ 75% negative responses for items written negatively. Similarly, “fragile areas of patient safety” that require improvement are those with ≤ 50% positive responses written positively or ≤ 50% negative responses for items written negatively. The average of all items within a dimension of patient safety culture was calculated to determine the dimensional score.

The hospitals in this study were categorized into two groups: accredited hospitals (AH) and non-accredited hospitals (NAH), based on their accreditation status during the study period. To achieve accreditation, hospitals underwent an evaluation by an independent accrediting institution, in a voluntary process initiated by the hospitals themselves. Accredited hospitals were certified by one of the following organizations: Joint Commission International (JCI) [17], *Qmentum International* (Accreditation Canada) [18], or *ONA* (‘Organização Nacional de Acreditação’, i.e., National Accreditation Organization) [19]. While JCI and *Qmentum International* are international accreditation systems, ONA is a nationally recognized accreditation body in Brazil. Each organization establishes quality and safety standards for hospital operations, but their frameworks and assessment methodologies differ.

For the final analysis, we exclusively considered fully completed questionnaires. The survey response rate was calculated by dividing the number of completed surveys by the total number of distributed surveys. All professionals were invited to participate, and recruitment was facilitated by each hospital’s patient safety centre coordinators, who disseminated an electronic survey link through internal communication channels. This report adheres to the Checklist for Reporting Results of Internet E-Surveys (CHERRIES) guidelines [20].

### Statistical analysis

Data were entered into Excel spreadsheets using a double-entry method, and a comprehensive review was conducted to detect and rectify any discrepancies. Descriptive statistics were used to present the frequencies of sample characteristics and patient safety climate factors. Negatively worded items were inverted to ensure that positive responses indicated a higher score. Consequently, a higher score signified a more positive attitude among healthcare workers towards patient safety culture.

A linear mixed-effects regression model was applied to analyze trends in patient safety culture over time and assess its association with hospital accreditation. This model was selected due to the hierarchical structure of the data, allowing for the inclusion of fixed effects to estimate the overall temporal trend and the difference between accredited and non-accredited hospitals, while random effects accounted for hospital-level variability and within-hospital correlations across time points [21]. Differences between groups (AH vs. NAH) in dimension scores are presented as point estimates along with 95% confidence intervals (CI). The magnitude of the effects was defined by the Hodges-Lehmann method to estimate the median difference (MD) and its 95% CIs between the groups. Scores for AH and NAH were compared using the Mann-Whitney test. All analyses were conducted using R software, version 4.1.2 (R Foundation for Statistical Computing). Statistically significant results were indicated by P-values less than 0.05.

## Results

In total, 259,268 responses were analysed, of which 70.8% were from the Southeast region, 19.8% from the Northeast, 8.5% from the Central-West, 0.8% from the South, and 0.2% from the North. In addition, 146,628 (57.2%) were full-time employees, and 80,843 (31.5%) had been working in their respective organizations for five years or more. Overall, physicians accounted for 26,925 (14%) of the respondents, while nursing staff accounted for 32,685 (17%). Additionally, nursing technicians represented 44% of the respondents, pharmacy staff accounted for 3%, nutrition staff 2%, and administrative professionals 8% (Supplementary Fig. 1). The represented work units included inpatient units (18.0%; *n* = 40,400), intensive care units (19.9%; *n* = 28,994), surgical and obstetric centres (13.0%; *n* = 29,192), emergency units (12.9%; *n* = 28,994), and other units (36.2%; *n* = 93,855) (Supplementary Fig. 1). Of the hospitals analyzed, 16 (22.5%) remained without accreditation throughout the historical series examined. Hospitals that achieved accredited status over time represented 18% (13 hospitals) of the total. All accredited hospitals were reaccredited in a timely manner and in accordance with the applied methodologies. In accredited hospitals, the overall response rate was 89%, whereas in non-accredited hospitals, it was 84% (Supplementary Table 1).

**Fig. 1:**
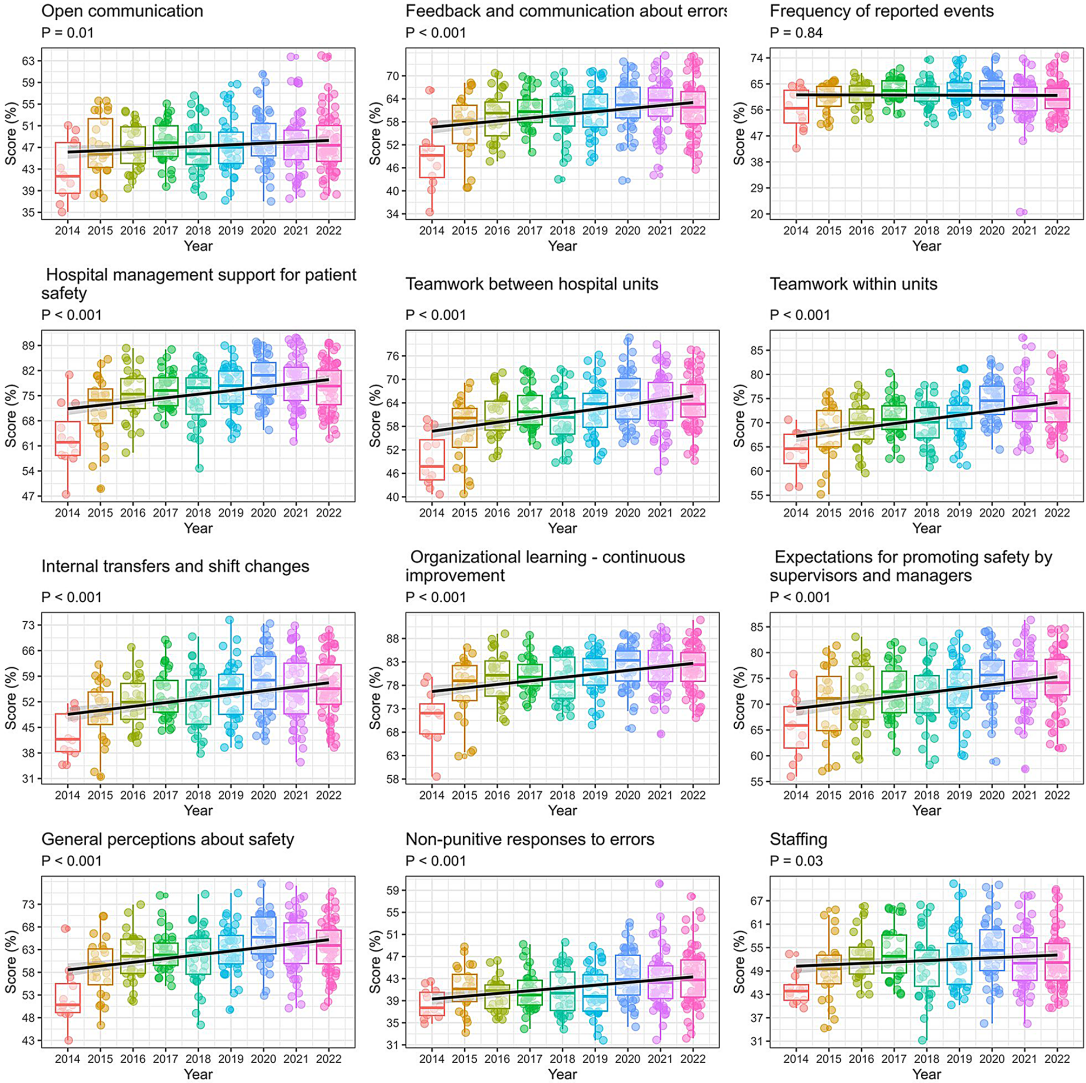
Hospital survey on patient safety culture dimensional scores over time from 2014 to 2022. Boxplot: Median (25–75% percentile) of scores by year for each dimension. The median marks the mid-point of the data and is shown by the line that divides the box into two parts. The upper and lower whiskers represent scores outside the middle 50% (i.e. the lower 25% of scores and the upper 25% of scores). Outliers are shown as circles. A linear mixed-effects regression model was applied to fit the trend for the dimension score over time

Overall, out of the 12 measured dimensions in the HSOPSC Survey, 11 showed significant improvement over an 8-year period (*p* < 0.05) (Fig. 1). The only dimension that did not show significant improvement was “frequency of reported events”, which remained unchanged over time (*p* = 0.84).

Table 1 presents the median differences in patient safety dimensional scores from the Hospital Survey on Patient Safety Culture (HSOPSC) between non-accredited and accredited hospitals. Overall, “Management support for patient safety” (77.35% [59.09–89.25]), and “organizational learning” (81.23% [69.23–88.95]) received positive responses from at least 75% of those surveyed, earning the distinction as “strong areas of patient safety”. Both AH and NAH exhibited similar areas of strengths and weaknesses. The median difference scores of the 42 questions in the HSOPSC survey between AH and NAH are shown in Supplementary Table 2.

**Table 1:** Median difference of the hospital survey on patient safety culture (HSOPSC) patient safety dimensional score between non-accredited and accredited hospitals

Dimensions	Dimensional Score (Overall)	Non-accredited Hospital Score*	Accredited Hospital Score*	Absolute Difference‡
				95%CI
1.Communication openness	47.13 [38.19–58.73]	46.9 [37.84–56.27]	47.28 [38.5–58.89]	0.68 (-0.73–2.2)
2.Feedback and communication about errors	61.45 [44–72.84]	60.3 [45.02–71.17]	61.54 [43.84–73.39]	1.53 (-0.47–3.59)
3. Frequency of events reported	61.28 [50.17–72.23]	60.54 [47.98–69.98]	61.59 [51.2–72.46]	1.5 (-0.01–2.99)
4. Management support for patient safety	77.35 [59.09–89.25]	77.22 [62.88–88.73]	77.37 [58.42–89.54]	0.35 (-1.82–2.4)
5. Teamwork across units	62.65 [46.55–75.78]	64.34 [49.27–75.81]	62.55 [46.46–75.59]	-1.93 (-4–0.31)
6. Teamwork within units	71.78 [61.23–81.69]	73.03 [64.57–81.83]	71.67 [60.94–81.46]	-1.29 (-2.69–0.12)
7. Handoffs and transitions	53.87 [38.99–69.9]	54.63 [40.99–70.85]	53.81 [38.49–69.36]	-1.55 (-4.34–1.08)
8. Organizational learning	81.23 [69.23–88.95]	80.39 [69.61–88.81]	81.33 [69.43–88.9]	0.76 (-0.71–2.27)
9. Supervisor/manager expectations and actions promoting safety	73.3 [59.29–83.85]	75.67 [62.94–84.03]	73.29 [59.24–82.98]	-2.42 (-4.28– -0.4)
10. Overall perception of patient safety	63.15 [50.09–73.93]	61.47 [49.48–76.26]	63.34 [50.58–72.47]	1.86 (0.02–3.76)
11. Nonpunitive response to errors	41.24 [34.13–51.98]	40.92 [32.6–51.33]	41.25 [35.25–52.23]	0.82 (-0.5–2.14)
12. Staffing	51.67 [39.58–68.29]	52.87 [39.65–69.52]	51.48 [39.55–66.08]	-2.07 (-4.28–0.11)

*Values expressed as Median [percentile 25–75%]; ‡Median difference (95% confidence intervals (CI)) estimated by the Hodges-Lehmann method

**Fig. 2:**
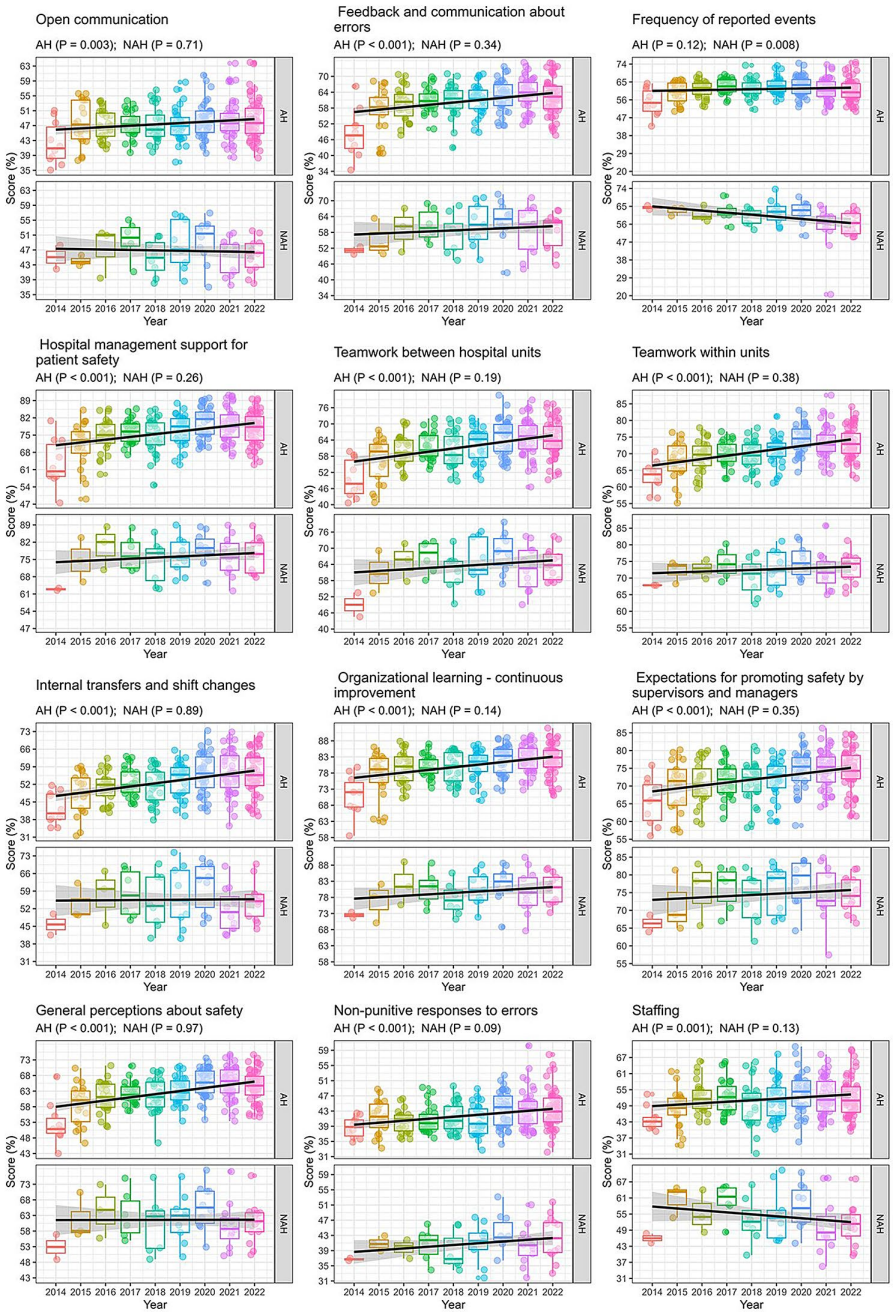
Comparison of the hospital survey on patient safety culture dimensional scores between accredited and non-accredited hospitals over time from 2014 to 2022. Boxplot: Median (25–75% percentile) of scores by year for each dimension. The median marks the mid-point of the data and is shown by the line that divides the box into two parts. The upper and lower whiskers represent scores outside the middle 50% (i.e. the lower 25% of scores and the upper 25% of scores). Outliers are shown as circles. A linear mixed-effects regression model was applied to fit the trend for the dimension score over time. NAH: non-accredited hospital; AH: accredited hospital

Over time, AH exhibited a statistically significant increase in safety culture across 11 of the 12 analysed dimensions, except for “frequency of reported events” (*p* = 0.12), which remained unchanged. In NAH, “frequency of reported events” decreased over time (*p* = 0.008), and the other dimensions showed no significant change (Fig. 2).

**Fig. 3:**
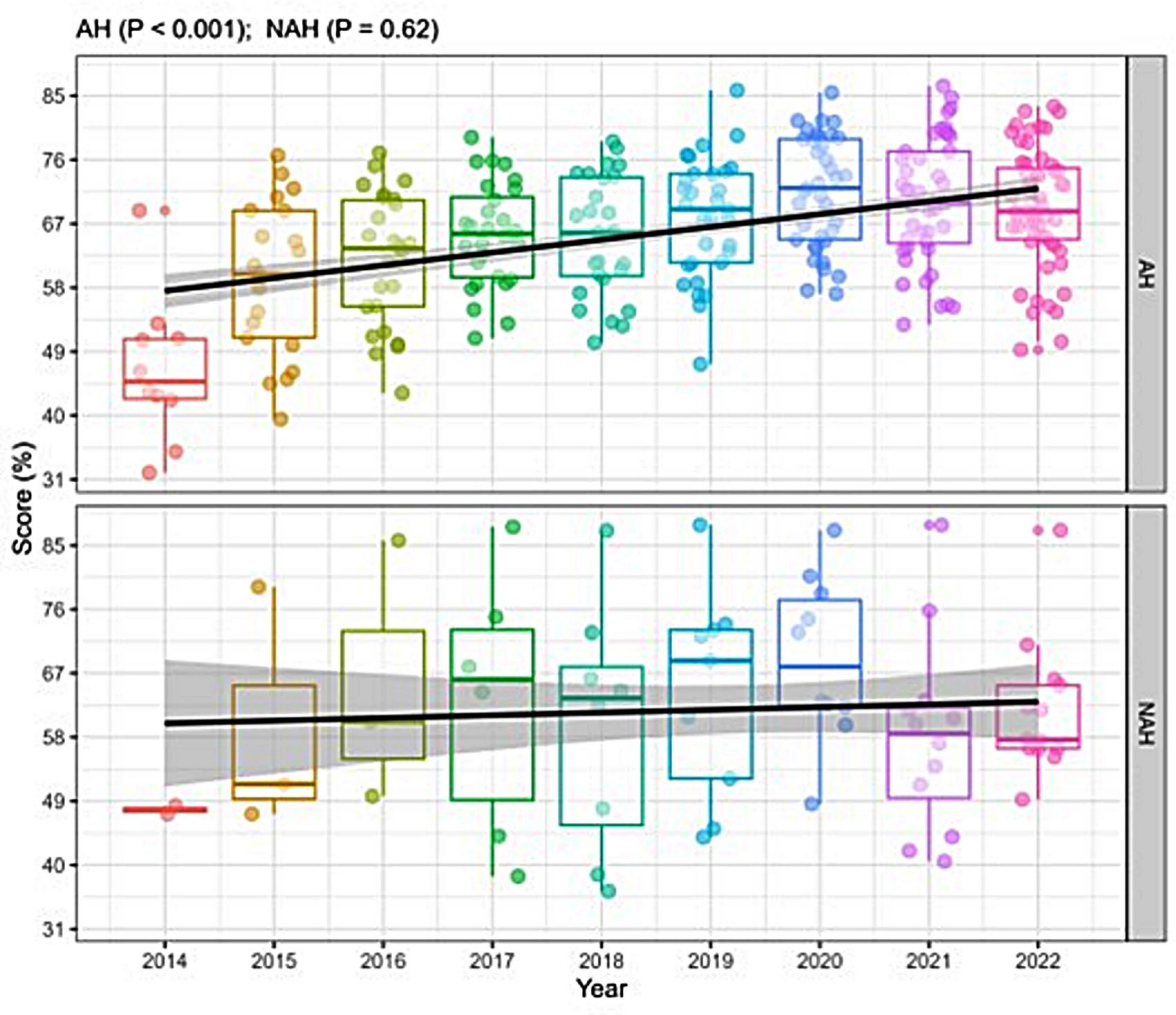
Patient safety rating as “very good” or “excellent” between accredited and non-accredited hospitals over time from 2014 to 2022. Boxplot: Median (25–75% percentile) of scores by year for each dimension. The median marks the mid-point of the data and is shown by the line that divides the box into two parts. The upper and lower whiskers represent scores outside the middle 50% (i.e. the lower 25% of scores and the upper 25% of scores). Outliers are shown as circles. A linear mixed-effects regression model was applied to fit the trend for the dimension score over time. NAH: non-accredited hospital; AH: accredited hospital

The assessment of patient safety as “very good” or “excellent” demonstrated a significant upward trajectory over time in the AH group (*p* < 0.001), whereas no statistically significant positive trend was observed in the NAH group (*p* = 0.62) (Fig. 3). The percentage of professionals who reported having submitted at least one event notification in the past 12 months was 46.38% [24.83–65.86] in NAH and 54.9% [34.23–71.91] in AH; MD 8.21 [95% CI 5.1-11.29]) (Supplementary Table 1). An additional exploratory analysis was conducted to evaluate whether the type of accrediting body—national (ONA) versus international (JCI or *Qmentum International*)—was associated with differences in healthcare professionals’ perceptions of patient safety culture. Dimensional scores from the HSOPSC survey were compared across hospitals grouped by accreditation framework. No statistically significant or clinically relevant differences were observed between hospitals accredited by national and those accredited by international bodies across any of the twelve safety culture dimensions. The detailed results of this comparative analysis are presented in Supplementary Table 4.

## Discussion

Patient safety culture is the combination of individual and collective values, attitudes, and behavioural patterns that influence a healthcare organization’s commitment to, and the accomplishment of, patient safety management [22]. Understanding the perceptions of healthcare professionals regarding safety culture raises awareness, identifies strengths and weaknesses, and monitors trends and impact. Our study observed a positive trend in the improvement over time of (and a correlation between hospital association with) the perception (by healthcare workers) of enhanced patient safety culture.

The studies conducted within the Brazilian public healthcare system revealed a perception of a fragile patient safety culture across the majority of the dimensions analysed [4, 5]. Different results were demonstrated in the private healthcare system assessed in our study, as almost all dimensions of patient safety culture improved over time, and only two remained as fragile areas of patient safety by the end of the analysed period. These observed differences between the public and private hospitals, against the background of major discrepancies in access to and the quality of healthcare between publicly and privately funded healthcare, warrant further exploration [4, 5].

Management perception has consistently been identified as one of the domains with lower scores in studies in Brazil and other countries [4, 23, 24]. In this study, despite the dimension related to “hospital management support for patient safety” being a strong point, “open communication” scores remained < 50%, the statistically significant improvement in perception over time notwithstanding. Communication openness reflects the team members’ ability to question the decisions and actions of individuals in higher authority when patient safety is at risk. The evidence suggests that hospital staff might not feel competent or comfortable enough to speak up. Our findings mirror the data from a U.S. survey of 447,584 hospital staff members, where 65% of respondents reported being afraid to ask questions when they sensed something was wrong [25].

The dimension of “nonpunitive response to errors” received the lowest score in our study and showed the least overall progression over time. This finding aligns with previous research that had identified this dimension as a challenge in patient safety management and promotion [26]. Coupled with the lack of improvement in the “frequency of reported events” over time, this low score suggests that the fear of shame or reprisal when errors are made [27] needs to be addressed.

Despite > 50 years of history of hospital accreditation in the U.S. and 24 years in Brazil, there has been limited research on the association between accreditation status and measures of hospital quality or patient safety culture [28, 29]. Accreditation is a multifaceted intervention ideally customized across diverse healthcare settings and should be linked to improved performance at a specific moment, and to the rate of improvement over time. Our study is apparently the first to demonstrate the association of accreditation status with improvement in the trajectory of patient safety culture in South America.

One hypothesis examined in this investigation was whether professionals in accredited institutions, due to their involvement in a more standardised healthcare system, might possess a higher level of critical thinking and a better understanding of safety culture. Consequently, their perception of safety culture could be more critical and paradoxically be worse compared to non-accredited hospitals. However, this concern was not confirmed as the questionnaire results identified an improving trend over time in patient safety culture in AH and not in HA.

Several international healthcare organizations have deliberated on the effectiveness of using accreditation standards as a tool to enhance both organizational and clinical performance [30, 31]. Nevertheless, the available evidence supporting this assumption remains limited [29], and presents a nuanced perspective on its effects [32]. On one hand, positive effects of hospital accreditation have been demonstrated in organizational culture and patient safety systems [6, 33]. In a pre- and post-accreditation assessment of nurses in tertiary hospital, a slight but statistically significant enhancement was noted across all HSOPSC dimensions [34]. This result agrees with ours, although in our sample of all healthcare workers there was only a significant improvement in four dimensions in hospitals that underwent accreditation. In a Danish hospital, the accreditation process was found to have a positive influence on management priorities, including patient safety culture [35]. Lastly, an observational study involving nurses in South Korea reported a statistically significant association between patient safety culture and the accreditation exercise [36]. On the other hand, several reviews have reported insufficient evidence regarding the impact of accreditation on measurable changes in the quality of care, health outcomes, and patient satisfaction [7, 33, 34].

Our findings suggest that hospital accreditation is associated with improved perceptions of patient safety culture among healthcare professionals. However, as emphasized in previous research, changes in knowledge and attitudes do not necessarily translate into sustained behavioral modifications or improved clinical outcomes [37, 38]. A persistent challenge in accreditation processes is ensuring that observed improvements extend beyond formal compliance during evaluation periods and result in enduring shifts in professional practice. While accreditation may foster adherence to safety protocols and enhance awareness of patient safety principles, its direct influence on measurable outcomes—such as morbidity, mortality, and long-term behavioral change—remains inconclusive [9, 10]. Moreover, the discrepancy frequently observed between what professionals know, their reported attitudes toward safety, and their actual behaviours in daily clinical settings further complicates this interpretation. This underscores the need for future research to combine perceptual assessments with objective clinical metrics and qualitative methodologies—such as structured interviews or focus groups—to more comprehensively evaluate the real-world impact of accreditation. Importantly, the present study was designed to identify associations rather than causal relationships, and its findings should be interpreted within this analytical framework.

Although national and international accreditation frameworks differ in structure, scope, and emphasis, our additional analysis did not reveal meaningful differences in safety culture perceptions between hospitals accredited by ONA and those accredited by JCI or *Qmentum international*. These findings suggest that the accreditation process itself—rather than the specific accrediting body—may be the critical driver in promoting improvements in patient safety culture. As all frameworks share core principles related to organizational commitment, standardization of practices, and continuous quality improvement, it is plausible that their implementation yields comparable effects on staff perceptions of safety. This observation supports the idea that consistent internal engagement with accreditation requirements may be more influential than the external label of the accrediting institution.

During the 8-year study period, multiple patient safety and quality improvement measures were systematically implemented across all hospitals participating in this study. Notably, as all hospitals belong to the same healthcare network, they operate under a unified quality management structure, ensuring consistent implementation of these initiatives network-wide over time. Key interventions included introducing a standardized Manual of Clinical Practices across all hospitals to unify quality and safety procedures, conducting regular audits accompanied by continuous feedback on performance outcomes, and reinforcing Patient Safety Units while cultivating a just culture environment to encourage transparent reporting of adverse events and near-misses. Furthermore, educational and motivational campaigns were uniformly deployed network-wide to stimulate greater event notification, supported by user-friendly electronic reporting systems. Regular monthly meetings were organized to share best practices, promoting benchmarking and continuous learning among hospital units. Additional unified measures included developing standardized guidelines aimed at improving patient experience, implementing a structured Professional Development Program, introducing checklists and standardized protocols for high-risk procedures, and conducting leadership rounds to recognize best practices and proactively address emerging safety concerns. Collectively, these integrated and consistently applied strategies were intended to maximize staff engagement, enhance interprofessional communication, and foster a network-wide organizational culture that prioritizes patient safety and quality of care.

This study has several potential limitations. Firstly, factors influencing accreditation decisions were not explicitly evaluated and may represent confounding variables, as hospitals already more motivated and committed to enhancing patient safety culture may be inherently more inclined to seek accreditation. Secondly, variations in healthcare professionals’ understanding of the questionnaire across units and over the extended study period introduce methodological challenges for accurate longitudinal comparisons. Thirdly, accreditation is only one element within broader performance improvement initiatives in healthcare, and potential interactions with concurrent quality and safety programs were not explored in this study. The effectiveness of accreditation likely depends on sustained institutional commitment and continuous monitoring beyond periodic assessment cycles. Fourthly, self-selection bias may have occurred due to differing response rates between accredited and non-accredited hospitals; specifically, lower participation rates from professionals in non-accredited hospitals could have resulted in overrepresentation of more safety-conscious respondents among accredited institutions, potentially inflating perceived accreditation benefits. Fifthly, our reliance on self-reported perceptions of patient safety culture is a significant limitation, as perceptual improvements captured through surveys do not necessarily correlate with actual behavioral changes or improved clinical outcomes. Future research should therefore incorporate objective behavioral assessments and clinical safety metrics to comprehensively evaluate the practical impact of accreditation processes. Lastly, the current study exclusively captured the perspectives of healthcare providers, indicating the need for future studies to include patient and stakeholder views, thus providing a more holistic assessment of patient safety culture.

## Conclusions

Our findings demonstrate a progressive enhancement in patient safety culture across a network of private hospitals in Brazil between 2014 and 2022. Accreditation was associated with a sustained promotion of safety culture; however, causality cannot be definitively inferred from our data. Notably, non-accredited hospitals reported fewer adverse events, a trend that may reflect underreporting rather than superior safety performance, thereby highlighting potential missed opportunities for system-wide learning and improvement. These observations underscore the critical role of fostering a robust reporting culture and suggest that accreditation, while valuable, must be complemented by broader organizational strategies aimed at continuously strengthening patient safety practices.

## Electronic supplementary material

Below is the link to the electronic supplementary material.

Supplementary Material 1

## Data Availability

The datasets generated during and/or analysed during the current study are available in Mendeley Data (Muniz da Silva, Leopoldo (2024), “Safety Culture Data.2024”, Mendeley Data, V1, DOI: 10.17632/p5zfv4ty3m.1). The files associated with this dataset are licensed under an attribution non-commercial 3.0 Unported license (CC BY NC 3.0).
