# Supplementary material for: Association between hospital accreditation and healthcare providers’ perceptions of patient safety culture: a longitudinal study in a healthcare network in Brazil
Source: Isr J Health Policy Res. 2025 Jun 4;14:27. doi: 10.1186/s13584-025-00690-8 (PMC12135554; doi:10.1186/s13584-025-00690-8)
Supplement: Supplementary file 1 — Supplementary Material 1 [file 13584_2025_690_MOESM1_ESM.docx]

**Improvements in patient Safety Culture:** **A National Survey in a PRIVATE healthcare network in Brazil**

**Electronic Supplementary Material**

Lima, et al.

| **Table of contents** | |  |
| --- | --- | --- |
| **Supplementary Files** | **Figure &Table Headings** | **Page** |
| **Supplementary Table 1.** | Number of questionnaires by year | **3** |
| **Supplementary Figure 1** | Responding professionals in the historical series (2014-2022). | **3** |
| **Supplementary Figure 2** | Distribution of responders by sector. Values expressed in absolute numbers and percentages. | **4** |
| **Supplementary Table 2** | Median difference of the Hospital Survey on Patient Safety Culture (HSOPSC) patient safety question scores between non-accredited and accredited hospitals. | **5** |
| **Supplementary Table 3** | Professionals who reported having submitted at least one event notification in the past 12 months: Median difference between non-accredited and accredited hospitals. | **7** |
| **Supplementary Table 4** | Comparison of Patient Safety Culture Dimension Scores by Type of Accreditation Body (National vs. International Accreditation). Accredited hospitals were certified by one of the following organizations: Joint Commission International (JCI), Qmentum International (Accreditation Canada) or ONA (‘Organização Nacional de Acreditação’, i.e., National Accreditation Organization). | **8** |
|  | **Checklist for Reporting Results of Internet E-Surveys (CHERRIES)** | **9** |

**Supplementary Table 1. Number of questionnaires by year**

| **Year** | **Effective number of questionnaires (Overall)** | **Response rate**  **(Overall)** | **Effective number of questionnaires**  **(Non-accredited hospitals)** | **Response rate**  **(Non-accredited hospitals)** | **Effective number of questionnaires**  **(Accredited hospitals)** | **Response rate**  **(Accredited hospitals)** |
| --- | --- | --- | --- | --- | --- | --- |
| 2014 | 6849 | 68.59 | 366 | 62.0 | 6483 | 69.0 |
| 2015 | 13318 | 92.57 | 756 | 86.0 | 12562 | 93.0 |
| 2016 | 23506 | 92.42 | 1377 | 84.0 | 22129 | 93.0 |
| 2017 | 26698 | 85.59 | 2628 | 82.0 | 24070 | 86.0 |
| 2018 | 27828 | 88.68 | 4444 | 87.0 | 23384 | 89.0 |
| 2019 | 32631 | 94.07 | 4796 | 89.0 | 27835 | 95.0 |
| 2020 | 36945 | 94.48 | 4619 | 91.0 | 32326 | 95.0 |
| 2021 | 43317 | 92.05 | 5500 | 86.0 | 37817 | 93.0 |
| 2022 | 48176 | 93.67 | 5105 | 91.0 | 43071 | 94.0 |

**Supplementary Figure 1.** Responding professionals in the historical series (2014-2022) (Percentage of responses by each professional category).

**Supplementary Figure 2.** Distribution of responders by sector. Values expressed in percentages.

**Supplementary Table 2.** Median difference of the Hospital Survey on Patient Safety Culture (HSOPSC) patient safety question scores between non-accredited and accredited hospitals.

| **Questions *** | **Overall** | **Non-accredited** | **Accredited** | **Median Difference**** |
| --- | --- | --- | --- | --- |
| 1. People support each other in this unit. | 69.35 [57.08 – 83.02] | 69.37 [60.39 – 82.3] | 69.34 [56.42 – 82.71] | -0.36 (-1.93 – 1.22) |
| 2. We have enough people to handle the workload. | 38.94 [21.74 – 65.28] | 40.41 [21.73 – 69.54] | 38.56 [22.49 – 63.89] | -2.34 (-5.58 – 1.03) |
| 3. When there is a lot of work to be done quickly, we work together as a team to accomplish the task. | 80.26 [70.16 – 88.84] | 81.94 [72.83 – 89.06] | 80.14 [69.96 – 88.57] | -2.08 (-3.5 – -0.61) |
| 4. In this unit, people treat each other with respect. | 83.15 [72.2 – 91.63] | 83.12 [72.31 – 90.98] | 83.15 [72.24 – 91.9] | -0.3 (-1.66 – 1.07) |
| 5. The employees in this unit work more hours than recommended in patient care. | 54.68 [39.42 – 71] | 58.29 [40.17 – 71.18] | 54.28 [39.21 – 70.17] | -3.08 (-5.38– -0.59) |
| 6. We are actively seeking improvements in patient safety. | 91.1 [79.62 – 96.42] | 90.74 [79.83 – 97.08] | 91.12 [79.95 – 96.23] | 0.08 (-1.11 – 1.27) |
| 7. We employ more temporary staff than recommended in patient care. | 65.67 [52.95 – 75.84] | 65.62 [49.75 – 76.63] | 65.67 [53.67 – 75.01] | -0.3 (-2 – 1.32) |
| 8. Employees feel that their mistakes are used against them. | 52.44 [43.02 – 67.63] | 53.47 [42.76 – 66.17] | 52.15 [43.41 – 67.99] | -0.64 (-2.46 – 1.23) |
| 9. Mistakes that occurred have led to positive changes in this unit. | 74.89 [60.83 – 84.74] | 73.29 [62.36 – 85.35] | 75.15 [60.65 – 84.38] | 1.09 (-0.65 – 2.85) |
| 10. It's only by chance that more serious errors don't occur here. | 60.51 [49.67 – 70.42] | 59.79 [46.88 – 71.49] | 60.57 [51.55 – 69.87] | 1.18 (-0.51 – 2.8) |
| 11. When one area in this unit gets very busy, the others pitch in to help. | 54.51 [40.4 – 69.13] | 56.51 [44.64 – 73.48] | 54.18 [38.72 – 68.77] | -2.59 (-4.65 – -0.66) |
| 12. When an event is reported, we feel that the focus is on the individual rather than the problem. | 50.5 [38.73 – 63.23] | 47.79 [38.12 – 59.77] | 50.76 [39.9 – 63.98] | 2.8 (0.99 – 4.55) |
| 13. When we make changes to improve patient safety, we assess their effectiveness. | 77.37 [65.47 – 86.81] | 76.14 [65.51 – 86.27] | 77.54 [65.67 – 86.97] | 0.95 (-0.73 – 2.65) |
| 14. We work in a "crisis mode," trying to do too much, too quickly. | 47.52 [33.77 – 65.44] | 49.49 [34.83 – 66.08] | 46.89 [33.58 – 65.14] | -3.63 (-6.3 – -0.93) |
| 15.Patient safety is never sacrificed in favor of working more. | 52.03 [40.26 – 63.42] | 52.86 [42.79 – 66.31] | 51.97 [39.86 – 61.5] | -1.65 (-3.57 – 0.11) |
| 16. Employees are concerned that their mistakes will be recorded in their personnel file. | 21.47 [15.04 – 30.85] | 21.38 [15.62 – 27.9] | 21.48 [14.92 – 31.16] | 0.48 (-0.61 – 1.53) |
| 17. We have issues with patient safety in this unit. | 68.52 [50.41 – 82.62] | 66.81 [50.03 – 84.8] | 68.98 [52.07 – 81.71] | 2.18 (-0.32 – 4.63) |
| 18.Our procedures and systems are effective at preventing errors from occurring. | 71 [50.63 – 83.42] | 66.67 [48.73 – 83.47] | 71.95 [53.52 – 83.39] | 5.16 (2.52 – 7.65) |
| 19. My supervisor/manager praises when they see work done in accordance with established procedures for patient safety. | 72.16 [54.85 – 84.09] | 75.33 [56.59 – 84.09] | 71.84 [54.96 – 84.06] | -3.12 (-5.33 – -0.9) |
| 20.My supervisor/manager takes employee suggestions for improving patient safety seriously. | 72.66 [56.02 – 85.02] | 74.5 [57.19 – 85.39] | 72.36 [55.84 – 84.12] | -2.98 (-5.21 – -0.76) |
| 21. Whenever the pressure increases, my supervisor/manager wants us to work faster, even if it means taking shortcuts. | 62.69 [49.24 – 75.42] | 64.67 [50.79 – 76.42] | 62.35 [48.83 – 74.7] | -2.76 (-4.76 – -0.68) |
| 22.My supervisor/manager ignores recurring patient safety issues. | 86.72 [76.1 – 93.35] | 87.21 [75.2 – 94.16] | 86.72 [76.62 – 92.73] | -0.88 (-2.21 – 0.57) |
| 23. We receive feedback on changes implemented based on reported events. | 53.85 [37.12 – 67.08] | 54.03 [37.8 – 67.08] | 53.85 [37.25 – 66.96] | 0.5 (-1.83 – 2.88) |
| 24. Employees voluntarily speak up if they see something that could negatively affect patient care. | 57.76 [45.61 – 70.73] | 56.4 [44.03 – 68.97] | 57.92 [45.96 – 71.31] | 1.63 (-0.2 – 3.53) |
| 25. We are informed about the errors that occur in this unit. | 60.12 [41.97 – 71.66] | 58.05 [43.01 – 68.67] | 60.52 [41.28 – 72.32] | 2.81 (0.85 – 4.77) |
| 26. Employees feel comfortable questioning decisions or actions of those in higher authority. | 33.33 [25.05 – 45.85] | 32.63 [22.73 – 43.72] | 33.33 [25.31 – 46.22] | 0.97 (-0.55 – 2.54) |
| 27. In this unit, we discuss ways to prevent errors from happening again. | 69.9 [53.28 – 81.41] | 68.71 [53.15 – 80.99] | 70.03 [55.07 – 81.35] | 1.65 (-0.49 – 3.87) |
| 28. Employees are hesitant to ask when something doesn't seem right. | 50 [42.18 – 62.15] | 51.75 [42.63 – 60.26] | 49.92 [41.92 – 62.56] | -0.36 (-1.85 – 1.15) |
| 29. How often are errors that are identified and corrected before they affect the patient reported? | 61.46 [50.27 – 72.24] | 61.24 [47.91 – 67.74] | 61.6 [50.89 – 72.28] | 1.4 (-0.12 – 2.88) |
| 30. How often are errors that have no potential patient harm reported? | 58.84 [47.97 – 71.27] | 58.52 [45.29 – 69.34] | 58.88 [48.16 – 71.55] | 1.38 (-0.19 – 2.86) |
| 31. How often are near-miss errors reported when they could have harmed the patient but did not? | 64 [52.27 – 73.91] | 62.63 [48.61 – 72.58] | 64.18 [53.68 – 73.94] | 1.84 (0.29 – 3.52) |
| 32. The hospital administration creates a work environment that promotes patient safety. | 83.47 [63.11 – 94.39] | 81.56 [65.72 – 94.9] | 83.81 [62.59 – 94.23] | 1.62 (-0.61 – 3.92) |
| 33. Hospital units do not coordinate well with each other. | 56.42 [39.99 – 72.14] | 57.14 [42.98 – 72.52] | 56.26 [39.58 – 71.77] | -1.07 (-3.58 – 1.28) |
| 34. Things "slip through the cracks" when patients are transferred from one unit to another. | 53.61 [38.76 – 70.41] | 55.09 [39.63 – 75.36] | 53.38 [38.75 – 69.68] | -2.53 (-5.29 – -0.1) |
| 35. There is good cooperation between hospital units that need to work together. | 62.61 [45.83 – 77.04] | 65.28 [47.79 – 77.09] | 62.32 [45.74 – 77] | -2.87 (-5.17 – -0.49) |
| 36.Critical information about patient care is lost during shift changes. | 51.97 [33.27 – 70.36] | 51.68 [35.67 – 71.48] | 52.15 [32.98 – 69.81] | -0.88 (-4.14 – 2.3) |
| 37. It is often unpleasant to work with employees from other hospital units. | 57.71 [42.79 – 70.92] | 60.31 [46.23 – 71.21] | 57.32 [42.55 – 69.19] | -2.56 (-4.43 – -0.71) |
| 38. Issues frequently arise in the exchange of information between hospital units. | 47.93 [33.44 – 64.52] | 48.75 [34.77 – 63.87] | 47.87 [33.42 – 64.38] | -1.2 (-3.53 – 1.13) |
| 39. The actions of the hospital administration demonstrate that patient safety is a top priority. | 81.82 [64.61 – 92.05] | 82.13 [68.38 – 91.67] | 81.73 [64.07 – 92.2] | -0.66 (-2.54 – 1.25) |
| 40. The hospital administration seems to be interested in patient safety only when an adverse event occurs. | 65.86 [49.13 – 81.6] | 66.24 [51.74 – 80.32] | 65.86 [48.99 – 82.2] | -0.23 (-2.67 – 2.15) |
| 41. The hospital units work well together to provide the best patient care. | 74.86 [53.88 – 86.66] | 74.85 [58.52 – 88.79] | 74.86 [53.51 – 86.35] | -0.96 (-3.34 – 1.34) |
| 42.Shift changes are problematic for patients in this hospital. | 62.14 [45.77 – 79.41] | 63.98 [48.61 – 80.36] | 61.9 [45.35 – 77.53] | -2 (-4.97 – 0.79) |

*Values expressed as Median [percentile 25-75%]; ** Median difference (95% confidence intervals - CI) estimated by the Hodges-Lehmann method.

**Supplementary Table 3.** Professionals who reported having submitted at least one event notification in the past 12 months: Median difference between non-accredited and accredited hospitals.

| **Questions *** | **Overall** | **Non-accredited** | **Accredited** | **Median Difference**** |
| --- | --- | --- | --- | --- |
| Professionals who reported having notified an event in the last 12 months. | 54.34 [29.91 – 71.66] | 46.38 [24.83 – 65.86] | 54.9 [34.23 - 71.91] | 8.21 (5.1 – 11.29) |
| Number of Event Notifications:  In the last 12 months, 1 to 2 event notifications. | 21.15 [15.43 – 27.22] | 19.72 [14.94 – 27.32] | 21.21 [15.85 – 26.9] | 1.24 (0.31 – 2.14) |
| Number of Event Notifications:  In the last 12 months, 3 to 5 event notifications. | 14.44 [7.13 – 20.07] | 13.05 [5.12 – 19.54] | 14.72 [7.65 – 20.08] | 2.01 (1.01 – 2.99) |
| Number of Event Notifications:  In the last 12 months, 6 to 10 event notifications. | 8.45 [2.94 – 15.14] | 6.87 [1.17 – 13.39] | 8.82 [3.96 – 15.21] | 2.08 (1.12 – 3.05) |
| Number of Event Notifications:  In the last 12 months, 11 to 20 event notifications. | 4.29 [1.19 – 9.94] | 2.82 [0.41 – 8.53] | 4.55 [1.39 – 10.1] | 1.44 (0.88 – 1.99) |
| Number of Event Notifications:  In the last 12 months, more than 20 event notifications. | 3.6 [0.64 – 10.76] | 2.1 [0 – 7.44] | 3.84 [0.99 – 11.55] | 1.58 (0.99 – 2.14) |

*Values expressed as Median [percentile 25-75%]; ** Median difference (95% confidence intervals - CI) estimated by the Hodges-Lehmann method.

**Supplementary Table 4.** Comparison of Patient Safety Culture Dimension Scores by Type of Accreditation Body (National vs. International Accreditation). Accredited hospitals were certified by one of the following organizations: Joint Commission International (JCI), Qmentum International (Accreditation Canada) or ONA (‘Organização Nacional de Acreditação’, i.e., National Accreditation Organization).

| **Dimensions *** | **Joint Commission International** | **National Accreditation Organization** | ***Qmentum International*** | **P-value** |
| --- | --- | --- | --- | --- |
| 1.Communication openness | 55.69 [48.48 - 65.22] | 57.57 [50.85 - 67.03] | 58.29 [49.96 - 65.99] | 0.09 |
| 2.Feedback and communication about errors | 60.24 [41.33 - 73.34] | 61.65 [47.73 - 71.76] | 62.45 [45.81 - 73.19] | 0.12 |
| 3. Frequency of events reported | 60.13 [50.41 - 72.57] | 61.28 [51.48 - 72.41] | 63.84 [52.3 - 71.81] | 0.08 |
| 4. Management support for patient safety | 76.54 [59.54 - 89.98] | 77.07 [59.15 - 88.94] | 79.75 [57.66 - 89.57] | 0.4 |
| 5. Teamwork across units | 60.35 [47.59 - 74.58] | 62.9 [46.51 - 76.88] | 62.79 [45.66 - 74.14] | 0.09 |
| 6. Teamwork within units | 69.69 [59.61 - 80.48] | 71.86 [61.7 - 82.05] | 71.84 [59.87 - 80.85] | 0.07 |
| 7. Handoffs and transitions | 53.72 [41.01 - 69.06] | 53.76 [38.54 - 69.24] | 55.37 [36.69 - 70.02] | 0.5 |
| 8. Organizational learning | 79.39 [67.83 - 88.71] | 81.58 [70.85 - 88.71] | 82.64 [67.8 - 89.25] | 0.09 |
| 9. Supervisor/manager expectations and actions promoting safety | 71.96 [58.25 - 82.64] | 73.68 [63.97 - 83.77] | 72.48 [58.53 - 81.47] | 0.07 |
| 10. Overall perception of patient safety | 63.35 [52.36 - 73.41] | 62.85 [50.45 - 72.11] | 65.4 [51.01 - 72.48] | 0.06 |
| 11. Nonpunitive response to errors | 38.96 [35.61 - 52.72] | 41.63 [34.87 - 52.41] | 41.82 [35.46 - 49.96] | 0.08 |
| 12. Staffing | 49.73 [41.6 - 61.54] | 50.99 [39.47 - 65.98] | 52.41 [39.92 - 68.34] | 0.06 |

*Values expressed as Median [percentile 25-75%] – Kruskal-Wallis test.

**Improvements in patient Safety Culture:** **A National Survey in a PRIVATE healthcare network in Brazil**

**Checklist for Reporting Results of Internet E-Surveys (CHERRIES)**

| ***Checklist Item*** | ***Explanation*** | ***Page Number*** |
| --- | --- | --- |
| Describe survey design | Describe target population, sample frame. Is the sample a convenience sample? (In “open” surveys this is most likely.) | 10-13 |
| IRB approval | Mention whether the study has been approved by an IRB. | 13 |
| Informed consent | Describe the informed consent process. Where were the participants told the length of time of the survey, which data were stored and where and for how long, who the investigator was, and the purpose of the study? | 13 |
| Data protection | If any personal information was collected or stored, describe what mechanisms were used to protect unauthorized access. | 11 |
| Development and testing | State how the survey was developed, including whether the usability and technical functionality of the electronic questionnaire had been tested before fielding the questionnaire. | 10-13 |
| Open survey versus closed survey | An “open survey” is a survey open for each visitor of a site, while a closed survey is only open to a sample which the investigator knows (password-protected survey). | 10-13 |
| Contact mode | Indicate whether or not the initial contact with the potential participants was made on the Internet. (Investigators may also send out questionnaires by mail and allow for Web-based data entry.) | 10-13 |
| Advertising the survey | How/where was the survey announced or advertised? Some examples are offline media (newspapers), or online (mailing lists – If yes, which ones?) or banner ads (Where were these banner ads posted and what did they look like?). It is important to know the wording of the announcement as it will heavily influence who chooses to participate. Ideally the survey announcement should be published as an appendix. | 10-13 |
| Web/E-mail | State the type of e-survey (eg, one posted on a Web site, or one sent out through e-mail). If it is an e-mail survey, were the responses entered manually into a database, or was there an automatic method for capturing responses? | 11-12 |
| Context | Describe the Web site (for mailing list/newsgroup) in which the survey was posted. What is the Web site about, who is visiting it, what are visitors normally looking for? Discuss to what degree the content of the Web site could pre-select the sample or influence the results. For example, a survey about vaccination on a anti-immunization Web site will have different results from a Web survey conducted on a government Web site | 11-12 |
| Mandatory/voluntary | Was it a mandatory survey to be filled in by every visitor who wanted to enter the Web site, or was it a voluntary survey? | 11-12 |
| Incentives | Were any incentives offered (eg, monetary, prizes, or non-monetary incentives such as an offer to provide the survey results)? | NA |
| Time/Date | In what timeframe were the data collected? | 10-12 |
| Randomization of items or questionnaires | To prevent biases items can be randomized or alternated. | NA |
| Adaptive questioning | Use adaptive questioning (certain items, or only conditionally displayed based on responses to other items) to reduce number and complexity of the questions. | NA |
| Number of Items | What was the number of questionnaire items per page? The number of items is an important factor for the completion rate. | 10-12 |
| Number of screens (pages) | Over how many pages was the questionnaire distributed? The number of items is an important factor for the completion rate. | 10-12 |
| Completeness check | It is technically possible to do consistency or completeness checks before the questionnaire is submitted. Was this done, and if “yes”, how (usually JAVAScript)? An alternative is to check for completeness after the questionnaire has been submitted (and highlight mandatory items). If this has been done, it should be reported. All items should provide a non-response option such as “not applicable” or “rather not say”, and selection of one response option should be enforced. | 11 |
| Review step | State whether respondents were able to review and change their answers (eg, through a Back button or a Review step which displays a summary of the responses and asks the respondents if they are correct). | 11 |
| Unique site visitor | If you provide view rates or participation rates, you need to define how you determined a unique visitor. There are different techniques available, based on IP addresses or cookies or both. | NA |
| View rate (Ratio of unique survey visitors/unique site visitors) | Requires counting unique visitors to the first page of the survey, divided by the number of unique site visitors (not page views!). It is not unusual to have view rates of less than 0.1 % if the survey is voluntary. | NA |
| Participation rate (Ratio of unique visitors who agreed to participate/unique first survey page visitors) | Count the unique number of people who filled in the first survey page (or agreed to participate, for example by checking a checkbox), divided by visitors who visit the first page of the survey (or the informed consents page, if present). This can also be called “recruitment” rate. | NA |
| Completion rate (Ratio of users who finished the survey/users who agreed to participate) | The number of people submitting the last questionnaire page, divided by the number of people who agreed to participate (or submitted the first survey page). This is only relevant if there is a separate “informed consent” page or if the survey goes over several pages. This is a measure for attrition. Note that “completion” can involve leaving questionnaire items blank. This is not a measure for how completely questionnaires were filled in. (If you need a measure for this, use the word “completeness rate”.) | NA |
| Cookies used | Indicate whether cookies were used to assign a unique user identifier to each client computer. If so, mention the page on which the cookie was set and read, and how long the cookie was valid. Were duplicate entries avoided by preventing users access to the survey twice; or were duplicate database entries having the same user ID eliminated before analysis? In the latter case, which entries were kept for analysis (eg, the first entry or the most recent)? | NA |
| IP check | Indicate whether the IP address of the client computer was used to identify potential duplicate entries from the same user. If so, mention the period of time for which no two entries from the same IP address were allowed (eg, 24 hours). Were duplicate entries avoided by preventing users with the same IP address access to the survey twice; or were duplicate database entries having the same IP address within a given period of time eliminated before analysis? If the latter, which entries were kept for analysis (eg, the first entry or the most recent)? | NA |
| Log file analysis | Indicate whether other techniques to analyze the log file for identification of multiple entries were used. If so, please describe. | NA |
| Registration | In “closed” (non-open) surveys, users need to login first and it is easier to prevent duplicate entries from the same user. Describe how this was done. For example, was the survey never displayed a second time once the user had filled it in, or was the username stored together with the survey results and later eliminated? If the latter, which entries were kept for analysis (eg, the first entry or the most recent)? | NA |
| Handling of incomplete questionnaires | Were only completed questionnaires analyzed? Were questionnaires which terminated early (where, for example, users did not go through all questionnaire pages) also analyzed? | 11 |
| Questionnaires submitted with an atypical timestamp | Some investigators may measure the time people needed to fill in a questionnaire and exclude questionnaires that were submitted too soon. Specify the timeframe that was used as a cut-off point, and describe how this point was determined. | NA |
| Statistical correction | Indicate whether any methods such as weighting of items or propensity scores have been used to adjust for the non-representative sample; if so, please describe the methods. | NA |

This checklist has been modified from Eysenbach G. Improving the quality of Web surveys: the Checklist for Reporting Results of Internet E-Surveys (CHERRIES). J Med Internet Res. 2004 Sep 29;6(3):e34 [erratum in J Med Internet Res. 2012; 14(1): e8.]. Article available at [https://www.jmir.org/2004/3/e34](https://www.jmir.org/2004/3/e34/)/; erratum available <https://www.jmir.org/2012/1/e8/>.
